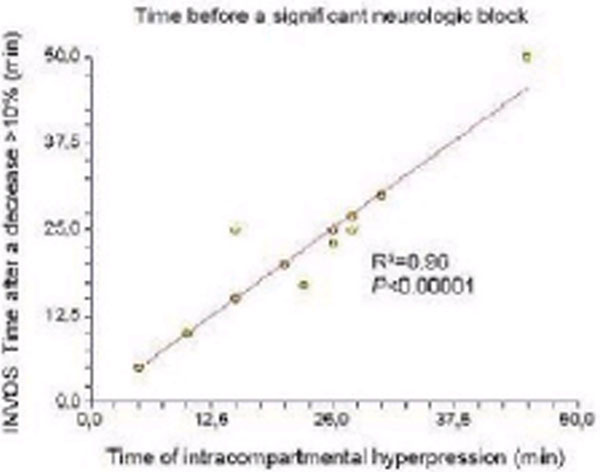# Muscular compartment syndrome and *in vivo *optical spectroscopy monitoring: a new model

**DOI:** 10.1186/cc9868

**Published:** 2011-03-11

**Authors:** F Ponchon, P Forget, M Vanhoonacker, G Stoquart, T Lejeune, F Lois, D Kahn, M De Kock

**Affiliations:** 1 UCL, Brussels, Belgium

## Introduction

The muscular compartment syndrome (MCS) is consecutive to an increase in intramuscular compartment pressures [1].This is a rare but serious postoperative complication. *In vivo *optical spectroscopy (INVOS) monitors tissular oxygenation continuously and non-invasively. Our objective was to develop a model mimicking the physiopathology of MCS and to assess the interest of the INVOS in this case [1-3].

## Methods

After approval of the ethics committee, we inflated a tourniquet in nine healthy volunteers at a pressure equal to the mean arterial pressure (MAP), obtaining a model of slight venous congestion and arterial hypoperfusion. The INVOS monitoring was compared with sensory deficits, pain, motor activity, electromyography and invasive pressure.

## Results

**Figure 1:**
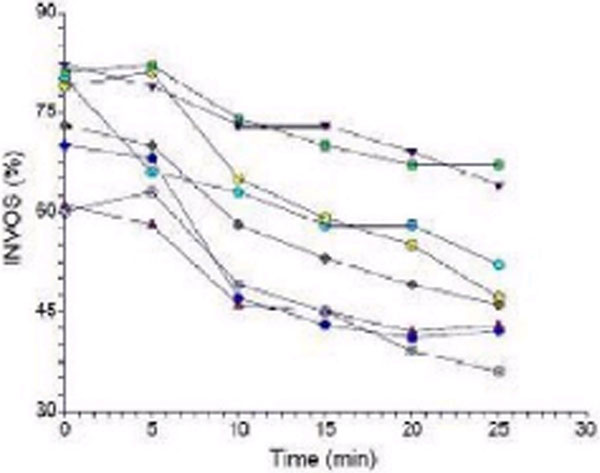


A profound motor nerve conduction block (>30% decrease in action potential amplitude from baseline) was observed in the seven volunteers completing the protocol, immediately reversible after releasing the external pressure. At baseline, the values of MAP, INVOS and intracompartmental pressure (ICP) were respectively 94.3 ± 6.5 mmHg, 73.3 ± 8.9% and 16.9 ± 8.6 mmHg. At the time of appearance of a significant block, the values of INVOS were 46.4 ± 10.9%; the absolute decrease of INVOS was 28.7 ± 10.6% and the ICP values were 70.0 ± 5.5 mmHg. The times to reach this significant block from baseline and from the time of an absolute INVOS decrease of 10% were respectively 33.0 ± 10.9 minutes and 27.43 ± 10.4 minutes (Figure 1).

## Conclusions

Our model is appropriate since it mimics perfectly MCS [3]. The time after achieving an absolute decrease of the INVOS value of 10% from baseline is as accurate as the time of intracompartmental hyperpression to predict MCS (Figure 2).

**Figure 2:**